# A Generalized Stress State and Temperature Dependent Damage Indicator Framework for Ductile Failure Prediction in Heat-Assisted Forming Operations

**DOI:** 10.3390/ma14175106

**Published:** 2021-09-06

**Authors:** Alan A. Camberg, Tobias Erhart, Thomas Tröster

**Affiliations:** 1 Chair of Automotive Lightweight Design, Faculty of Mechanical Engineering, Paderborn University, Mersinweg 7, 33100 Paderborn, Germany; thomas.troester@uni-paderborn.de; 2 DYNAmore GmbH, Industriestraße 2, 70565 Stuttgart, Germany; tobias.erhart@dynamore.de

**Keywords:** ductile failure, Forming Limit Curve, hot stamping, warm forming, sheet metal forming, heat-assisted forming, Johnson–Cook, deep-drawing, GISSMO, localized necking, forming limits

## Abstract

Heat-assisted forming processes are becoming increasingly important in the manufacturing of sheet metal parts for body-in-white applications. However, the non-isothermal nature of these processes leads to challenges in evaluating the forming limits, since established methods such as Forming Limit Curves (FLCs) only allow the assessment of critical forming strains for steady temperatures. For this reason, a temperature-dependent extension of the well-established GISSMO (Generalized Incremental Stress State Dependent Damage Model) fracture indicator framework is developed by the authors to predict forming failures under non-isothermal conditions. In this paper, a general approach to combine several isothermal FLCs within the temperature-extended GISSMO model into a temperature-dependent forming limit surface is investigated. The general capabilities of the model are tested in a coupled thermo-mechanical FEA using the example of warm forming of an AA5182-O sheet metal cross-die cup. The obtained results are then compared with state of the art of evaluation methods. By taking the strain and temperature path into account, GISSMO predicts greater drawing depths by up to 20% than established methods. In this way the forming and so the lightweight potential of sheet metal parts can by fully exploited. Moreover, the risk and locus of failure can be evaluated directly on the part geometry by a contour plot. An additional advantage of the GISSMO model is the applicability for low triaxialities as well as the possibility to predict the materials behavior beyond necking up to ductile fracture.

## 1. Introduction

Reducing vehicle weight offers remarkable opportunities for improving fuel economy and meeting global emissions regulations, regardless of the drivetrain concept [1]. Over the past years, high-strength materials have enabled a significant reduction in sheet thicknesses and have therefore established themselves as the preferred lightweighting approach for car body structures. However, high strength is usually associated with limited formability, which increases the demand for new manufacturing technologies that enable the drawing of high-strength and geometrically complex car body components. The ductility of both steel and aluminum sheet materials can be improved by warm and hot forming processes, which have been extensively investigated in recent years [2,3]. Although components are now manufactured in series by hot stamping [4], there is still a need for a generalized and robust simulation method to assess the forming limits of sheet metal under non-isothermal conditions in order to reduce try-out time and costs.

Since ductile fracture of sheet metals is often preceded by necking, the Forming Limit Diagram (FLD) asserted itself as an established post-processing approach for formability studies in finite element (FE) simulations of conventional stamping processes [5]. The Forming Limit Curve (FLC), as a failure criterion for FLD studies, is usually determined using an experimental method proposed by Nakazima et al. [6], which is described further in the ISO 12004-2:2021 standard [7]. To reduce the effort of experimental FLC determination, several theoretical models for FLC calculation have been introduced over the last six decades [8], including the widely established Marciniak–Kuczyński (M-K) model [9].

However, a single FLC represents the forming limit strains for only one specific test condition and is therefore not suitable for non-isothermal forming processes, as the material properties are strongly influenced by temperature and strain rate [10,11]. Consequently, multiple isothermal FLCs have to be determined at different elevated temperatures and strain rates to study the risk of necking in heat-assisted forming processes [12,13]. Even though several isothermal FLCs are available, the non-isothermal effects are often neglected due to the lack of generalized methods for evaluating the forming limits during warm and hot forming processes, and so the forming limits are investigated in analogy to cold forming with only one steady FLC (e.g., [14,15,16]).

An extension of a classical FLC by temperature seems to be a reasonable improvement for non-isothermal forming applications. As discussed by Krauer and Hora [17], a surface can be superimposed over several FLCs at different temperatures to obtain a temperature-dependent “Forming Limit Surface” for non-isothermal simulations. A similar approach was presented by Cui et al. [18], where a temperature-dependent 3D FLD was used to predict the forming limits in a direct press hardening process. However, a fundamental disadvantage of the 3D FLD approach is the neglect of non-linear strain and non-constant temperature paths of the material during hot stamping. As pointed out by Krauer and Hora [17] as well as by Gao et al. [19], the differential form of theoretical FLC models with thermal extensions can be implemented directly in FE routines, such that non-linearity in strain and temperature paths can be directly considered. The validity of that modeling approach has been demonstrated lately in the literature (e.g., [19,20]).

Currently, stress state dependent damage indicator frameworks have gained considerable interest to predict material behavior up to ductile fracture. The Generalized Incremental Stress State Dependent Damage Model (GISSMO) described by Andrade et al. [21] represents a generalized fracture indicator framework applicable for conventional yield locus functions and arbitrary fracture envelopes, which are straightforward and easy to calibrate from experimental tests. Furthermore, in lose analogy to the method proposed by Lemaitre [22], GISSMO allows material softening by coupling the damage to the stress tensor when a critical (local instability) strain is reached. The GISSMO fracture indicator framework combined with a suitable fracture envelope proved its feasibility to predict fracture in different sheet metal forming applications [23,24,25,26].

Compared to ductile fracture modeling at isothermal conditions, the literature shows significantly fewer approaches for non-isothermal processes. Lin et al. [27] introduced a Continuum Damage Mechanics (CDM) based model with temperature and strain rate effects to predict the formability at non-isothermal conditions which was successfully applied by Bai et al. [28] for warm forming. Johnson and Cook [29] introduced a phenomenological fracture model for ballistic problems which takes into account the effects of stress state, strain rate and temperature. However, the rudimentary fracture criterion and the linear fracture strain dependency on temperature in the initial Johnson–Cook (JC) model do not allow the necessary flexibility to precisely capture experimental data. Buyuk [30] disassembled the classical JC model to access a generalized multiplicative fracture model with arbitrary functions for stress state, temperature scaling, and strain rate dependency. Further enhancements of that model were introduced by Haight [31] and Sengoz [32]. As shown in a wealth of scientific publications, JC-based models are able to provide reliable predictions on fracture in high velocity ballistic impacts by using fine finite element models [30,31,32,33,34,35]. However, to the best of the authors’ knowledge, the JC-based modeling approach has not yet been successfully used to predict neither the onset of necking nor fracture in non-isothermal sheet metal forming simulations with shell elements.

In this work, a temperature extension of GISSMO is proposed to obtain a more general form of the tabulated model presented by Buyuk [30]. A key feature of the proposed fracture indicator framework is, unlike in the classical JC formulation, the comprehensive stress state and temperature dependency of the fracture strain. This is achieved by an extension of the mixed stress-strain space by a temperature dimension. As a result, a stress state, equivalent plastic strain and temperature dependent fracture surface is obtained. The same applies also for the instability strain which can be understood as a 3D FLC. After presenting the models’ backbone as a scalar damage indicator framework, material data adapted from Abedrabbo et al. [11] are used to test the model abilities in a warm forming process of a cross-die cup. Finally, the results are compared with a JC-like approach as well as a 3D FLD evaluation technique and discussed.

## 2. Materials and Methods

### 2.1. Material Data

The experimental and numerical data for an AA5182-O aluminum alloy sheet metal were adapted from Abedrabbo et al. [11]. By taking into account strain rate (0.001–0.08 s${}^{-1}$) and temperature (25–260 ${}^{\circ }$C) dependency on flow and anisotropy, all relevant material effects were considered. Based on the data shown in Table 1 and Table 2, a comprehensive constitutive modeling was carried out.

### 2.2. Plasticity

For the isotropic hardening a modified power law model initially introduced in [36] was used. The model takes into account the effects of strain rate sensitivity $m_{k}$ and temperature *T* on the flow stress *k*, where *K*, $n_{k}$, $m_{k}$ are material parameters dependent on temperature given in Table 1 and $\varepsilon_{0}$ is a constant value of 0.01

(1) $$k\left[{\bar{\varepsilon }}_{p},\dot{\varepsilon },T\right]=K\left[T\right]{\left({\bar{\varepsilon }}_{p}+\varepsilon_{0}\right)}^{n_{k}\left[T\right]}{\left(\dot{\varepsilon }\right)}^{m_{k}\left[T\right]}$$

whereby ${\bar{\varepsilon }}_{p}$ is the equivalent plastic strain and $\dot{\varepsilon }$ denotes the strain rate.

By assuming associated plastic flow, the anisotropy was described by the Barlat89-2D yield locus model proposed by Barlat and Lian [37]. The Barlat89-2D anisotropic equivalent stress ${\bar{\sigma }}_{Barlat89-2D}$ was defined as

(2) $${\bar{\sigma }}_{Barlat89-2D}=\frac{1}{2^{1/M}}\left(a\right|K_{1}+K_{2}{|}^{M}+a|K_{1}-K_{2}{|}^{M}+c|2K_{2}{{|}^{M})}^{1/M}$$

whereby *M* denotes the yield locus exponent which is assumed to be M=8, as given in the literature for fcc crystal structures [37]. The stress tensor invariants $K_{1}$ and $K_{2}$ were given by

(3) $$K_{1}=\frac{\sigma_{xx}+h\sigma_{yy}}{2}$$

(4) $$K_{2}=\sqrt{{\left(\frac{\sigma_{xx}-h\sigma_{yy}}{2}\right)}^{2}+p^{2}\sigma_{xy}^{2}}$$

where *a*, *c*, *h* and *p* are material parameters which can be obtained by the R-values to

(5) $$a=2-c=2-2\sqrt{\frac{r_{0}}{1+R_{0}}\cdot \frac{R_{90}}{1+R_{90}}}\;$$

(6) $$h=\sqrt{\frac{R_{0}}{1+R_{0}}\cdot \frac{1+R_{90}}{R_{90}}}\;$$

However, the parameter *p* could not be determined directly from $R_{0}$, $R_{45}$ and $R_{90}$ and was calculated iteratively by a numerical approximation method.

The R-values were provided as a function of temperature (Table 2), enabling a temperature dependent anisotropy modeling. Considering the effects of temperature and strain rate, the final yield condition read

(7) $$f\left[{\bar{\sigma }}_{Barlat89-2D},{\bar{\varepsilon }}_{p},\dot{\varepsilon },T\right]={\bar{\sigma }}_{Barlat89-2D}\left[\sigma^{*},T\right]-k\left[{\bar{\varepsilon }}_{p},\dot{\varepsilon },T\right]=0\;$$

where $\sigma^{*}$ denotes the Cauchy stress tensor.

**Figure 1:**
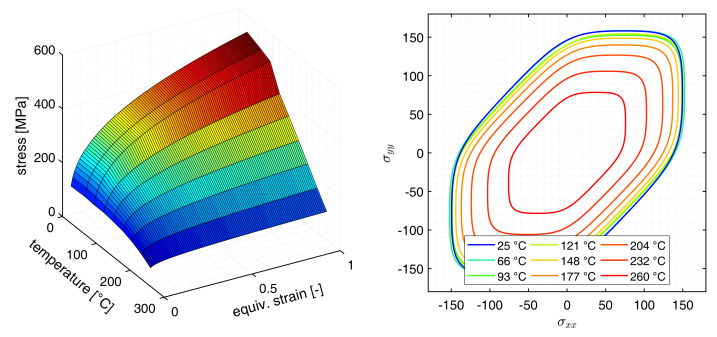
Temperature dependent hardening for $\dot{\varepsilon }$ = 0.01 s${}^{-1}$ and the Barlat89-2D temperature dependent yield locus for material parameters adapted from Abedrabbo et al. [11].

The resulting temperature dependent hardening as well as the temperature dependent yield locus are presented in Figure 1.

### 2.3. Forming Limit Curves

**Figure 2:**
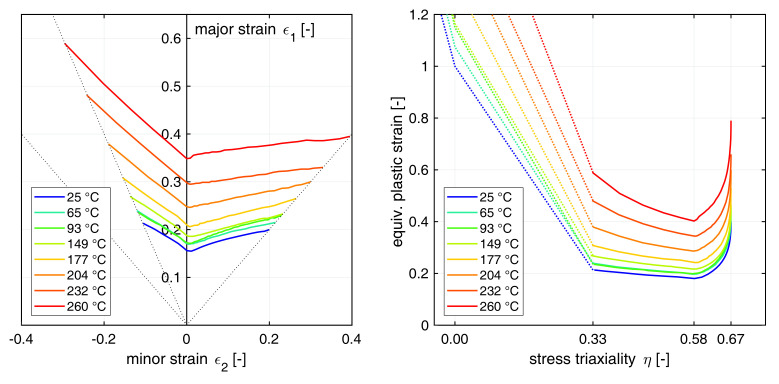
Temperature dependent FLCs for AA5182-O based on M-K analysis from Abedrabbo et al. [11]. (**Left**): FLCs depicted in the $\varepsilon_{1}-\varepsilon_{2}$ space; (**Right**): FLCs depicted in the $\eta -{\bar{\varepsilon }}_{p}$ space and extrapolated towards low triaxialities (dotted lines).

Several numerical M-K FLCs for isothermal conditions between 25 ${}^{\circ }$C and 260 ${}^{\circ }$C were adapted from Abedrabbo et al. [11] to describe the material forming limits. The FLCs are shown in the left part of Figure 2. By making use of the plane stress condition ($\sigma_{33}=0$), the forming limits were mapped from the principal strain to the mixed stress-strain space by relationships deduced by Lee [38]. In doing so, a direct transfer of the FLCs from the $\varepsilon_{1}-\varepsilon_{2}$ space into the $\eta -{\bar{\varepsilon }}_{p}$ space was enabled. The equations read

(8) $${\bar{\varepsilon }}_{p}=\varepsilon_{1}\frac{2}{\sqrt{3}}\sqrt{1+\alpha +\alpha^{2}}\;\;\;\mathrm{with}\;\;\;\alpha =\frac{\varepsilon_{2}}{\varepsilon_{1}}$$

and the stress state characterized by the stress triaxiality η for isochoric and associated plasticity as

(9) $$\eta =\frac{\sigma_{m}}{\bar{\sigma }}=\frac{I_{1}}{3\sqrt{3J_{2}}}=\frac{1}{3}\frac{\left(\sigma_{11}+\sigma_{22}\right)}{\sqrt{\sigma_{11}^{2}-\sigma_{11}\sigma_{22}+\sigma_{22}^{2}+3\sigma_{12}^{2}}}=\frac{1}{\sqrt{3}}\frac{1+\alpha }{\sqrt{1+\alpha +\alpha^{2}}}$$

where $I_{1}$ denotes the first invariant of the Cauchy stress tensor and $J_{2}$ denotes the second invariant of the deviatoric stress tensor. In other words, the stress triaxiality is defined as the ratio between the mean stress $\sigma_{m}$ and the equivalent (von Mises) stress $\bar{\sigma }$.

In addition, an experimental value for the equivalent plastic fracture strain at in-plane shear at 25 ${}^{\circ }$C of ${\bar{\varepsilon }}_{p}^{f}[\eta =0,T=25{\;}^{\circ }$C] = 1.0 was adapted from Rahmaan et al. [39] to take failure at low strain ratios/triaxialities into account. Furthermore, by setting ${\bar{\varepsilon }}_{p}^{f}[\eta =-1/3,T=25{\;}^{\circ }$C] = 3.0, it is assumed that no failure occurs under compression. Moreover, by assuming a stress-state independent proportionality between failure strains and elevated temperatures, as stated by Johnson and Cook [29], both values were scaled by the ratio of ${\bar{\varepsilon }}_{p}^{f}[\eta =2/3,T>25{\;}^{\circ }$C] ÷ ${\bar{\varepsilon }}_{p}^{f}[\eta =2/3,T=25{\;}^{\circ }$C] for elevated temperatures. The extended FLCs are depicted in the right part of Figure 2 in the mixed stress-strain space.

## 3. Damage Indicator Framework with Thermal Effects

In the pioneering work of Johnson and Cook [29] the damage variable *D* was defined as the accumulative ratio between the differential of equivalent plastic strain $d{\bar{\varepsilon }}_{p}$ and the equivalent plastic strain to fracture ${\bar{\varepsilon }}_{p}^{f}$ under the current conditions of stress state η, strain rate $\dot{\varepsilon }$ and temperature *T*

(10) $$D=\int \frac{d{\bar{\varepsilon }}_{p}}{{\bar{\varepsilon }}_{p}^{f}\left[\eta ,\dot{\varepsilon },T\right]}\;$$

For the incorporation of temperature and strain rate effects on the equivalent plastic strain to fracture, a phenomenological model with a multiplicative decomposition was proposed. The isothermal baseline fracture envelope is proportionally controlled by a strain rate and a temperature dependent term

(11) $${\bar{\varepsilon }}_{p}^{f}\left[\eta ,\dot{\varepsilon },T\right]=\left(D_{1}+D_{2}e^{D_{3}\eta }\right)\left(1+D_{4}\ln{\dot{\varepsilon }}^{*}\right)\left(1+D_{5}T^{*}\right)$$

where $D_{1}$, $D_{2}$, $D_{3}$, $D_{4}$ and $D_{5}$ are material parameters, ${\dot{\varepsilon }}^{*}$ is the normalized effective plastic strain rate and $T^{*}$ is the homologous temperature.

As pointed out by Johnson and Cook [29], the mathematical representation of the fracture envelope as an exponential function is not suitable to capture all experimental data sufficiently. To overcome this drawback Buyuk [30] disassembled the classical JC model into a product of three arbitrary functions, i.e., an isothermal baseline fracture envelope f[η], a strain rate $g[\dot{\varepsilon }]$ and a temperature h[T] dependent scaling function

(12) $${\bar{\varepsilon }}_{p}^{f}\left[\eta ,\dot{\varepsilon },T\right]=f\left[\eta \right]g\left[\dot{\varepsilon }\right]h\left[T\right]\;.$$

Although the model presented by Buyuk [30] allows an unlimited freedom in the mathematical formulation of its individual terms, the temperature dependence still has the same disadvantage as in the original JC model. By scaling the baseline fracture envelope by a stress state invariant expression, both models assume proportionality between failure strains and elevated temperatures. However, the opposite could be proven by numerous publications cited in Section 1. To overcome this drawback a temperature enrichment of the generalized stress state dependent fracture indicator framework GISSMO model is proposed here.

The GISSMO model is described in detail by Andrade et al. [21]. In the present work the scalar damage variable *D* as well as the instability variable *F* are enhanced by a temperature dependency. The equations read

(13) $$D={\left(\frac{{\bar{\varepsilon }}_{p}}{{\bar{\varepsilon }}_{p}^{f}\left[\eta ,T\right]}\right)}^{n}$$

(14) $$F={\left(\frac{{\bar{\varepsilon }}_{p}}{{\bar{\varepsilon }}_{p}^{ins}\left[\eta ,T\right]}\right)}^{n}$$

whereby the variables ${\bar{\varepsilon }}_{p}^{f}\left[\eta ,T\right]$ and ${\bar{\varepsilon }}_{p}^{ins}\left[\eta ,T\right]$ denote the equivalent plastic fracture strain and the equivalent plastic instability strain as arbitrary functions of the current stress triaxiality η and temperature *T*, respectively. In contrast to Equation (10), in the GISSMO approach a non-linear damage accumulation is introduced through the damage exponent *n*. Furthermore, in this current study the dependency of the equivalent strain to fracture on strain rate is neglected to establish comparability with the FLC approach. Calculating the derivatives of Equations (13) and (14)

(15) $$dD=n{\left(\frac{{\bar{\varepsilon }}_{p}}{{\bar{\varepsilon }}_{p}^{f}\left[\eta ,T\right]}\right)}^{n-1}\frac{d{\bar{\varepsilon }}_{p}}{{\bar{\varepsilon }}_{p}^{f}\left[\eta ,T\right]}$$

(16) $$dF=n{\left(\frac{{\bar{\varepsilon }}_{p}}{{\bar{\varepsilon }}_{p}^{ins}\left[\eta ,T\right]}\right)}^{n-1}\frac{d{\bar{\varepsilon }}_{p}}{{\bar{\varepsilon }}_{p}^{ins}\left[\eta ,T\right]}$$

and substituting their *n*-th root into Equations (15) and (16) respectively, yields the general expressions for damage *D* and instability *F*. By integrating both variables over the incremental development of plastic strain, the evolution of damage and instability follows as

(17) $$D=\int \frac{n}{{\bar{\varepsilon }}_{p}^{f}\left[\eta ,T\right]}D^{\left(1-1/n\right)}\;d{\bar{\varepsilon }}_{p}$$

(18) $$F=\int \frac{n}{{\bar{\varepsilon }}_{p}^{ins}\left[\eta ,T\right]}F^{\left(1-1/n\right)}\;d{\bar{\varepsilon }}_{p}$$

wherein fracture and instability are postulated to occur when the accumulated variables *D* and *F* reach a limiting value of one, respectively.

The capability of GISSMO to affect the stress tensor by damage is also available in the thermal extension in an unchanged formulation, i.e.,

(19) $$\sigma =\sigma^{*}\left[1-{\left(\frac{D-D_{ins}}{1-D_{ins}}\right)}^{m}\right]\;$$

Here, $\sigma^{*}$ denotes the undamaged Cauchy stress tensor and $D_{ins}{=D|}_{F=1}$, so the transition from a non-coupled to a coupled solution is assumed when the instability measure *F* reaches unity. That modeling technique is motivated by a material softening when localized necking occurs. Moreover, the so-called fading exponent *m* allows to control the coupling evolution. At this point, it should be mentioned that both the damage exponent *n* and the fading exponent *m* are assumed to be temperature invariant.

## 4. Coupled Thermo-Mechanical Finite Element Simulations of Warm Forming

**Figure 3:**
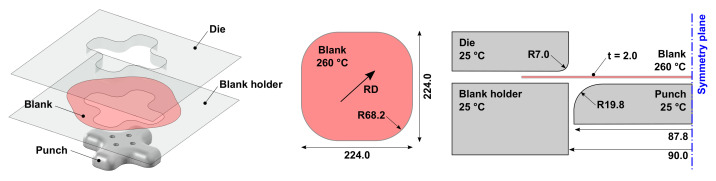
Geometries and initial temperatures of the cross-die cup tooling and blank. Length units are in [mm].

In order to evaluate the performance of the temperature-dependent GISSMO fracture indicator framework, the warm forming of a double symmetric cross-die presented in Figure 3 was numerically investigated and compared with an approach in analogy to Buyuk [30] and a 3D FLD approach as used in [18]. The fully coupled thermo-mechanical simulations were performed with LS-DYNA. In order to provide a realistic evaluation of the influence of the time-temperature history on the flow properties and the forming limits of the material, the model took into account thermal effects due to thermal conduction, thermal radiation and convection. The heat transfer between the mold and the blank took into account the pressure-dependent interfacial heat transfer coefficient $h_{IHTC}=f\left[p\right]$ given in Table 3. A constant value of μ=0.05 was selected for the coefficient of friction between the tool and the sheet metal blank as being the average value from tribological investigations on aluminum sheets at elevated temperatures from Noder et al. [40]. The blank sheet was modeled by four-node fully-integrated shell elements (element type 16 in LS-DYNA shell element library) with seven integration points through the thickness, an element edge length of $l_{el}=2.5$ mm and an initial temperature of $T_{blank}=260{\;}^{\circ }$C. The rolling direction (RD) was consistent with the diagonal of the blank sheet. The tooling had an initial temperature of $T_{tool}=25{\;}^{\circ }$C and was assumed to be rigid with a shell thickness of 10 mm to mimic the near-surface and thermally relevant volume of the dies. The blank holder force was set to $F_{BH}=50$ kN. The punch speed was $v_{punch}$ = 100 mm/s. Further model details are given in Table 4.

## 5. Results

### 5.1. Temperature Dependent 3D Forming Limit Diagram

In this section the risk of necking is evaluated in analogy to the temperature dependent 3D FLD method presented by Cui et al. [18]. By a linear interpolation between the isothermal FLCs a surface in the space of major strain $\varepsilon_{1}$, minor strain $\varepsilon_{2}$ and temperature *T* were created. In each time step it was evaluated whether the element values exceeded the 3D FLC or not. For the purpose of evaluation, the nodal temperatures were averaged over the element domain.

**Figure 4:**
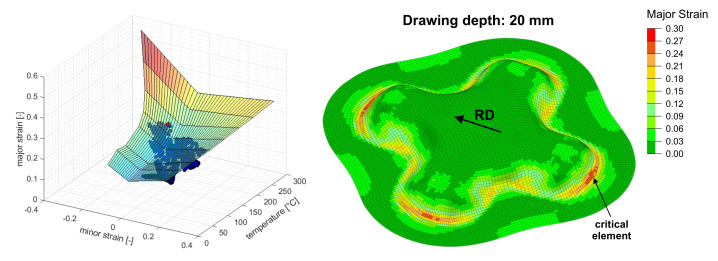
(**Left**): 3D FLC represented as a limiting surface with depicted element loading points at the predicted critical drawing depth; a critical element (red) exceeds the surface. (**Right**): Contour plot of the major strain at the inner surface of the deep-drawn cross-die cup at the predicted onset of necking.

**Figure 5:**
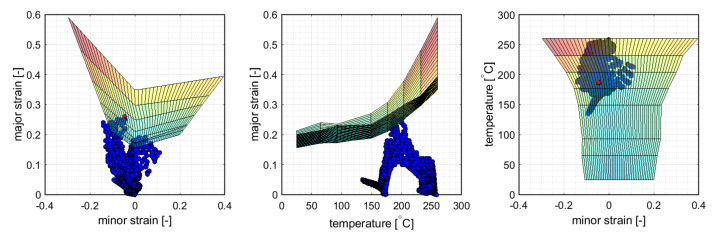
The 3D FLC at the predicted onset of necking from Figure 4 depicted in different views.

As shown in Figure 4, the onset of necking was predicted in rolling direction at the ends of the cross-die arms at a drawing depth of 20 mm. The corresponding 3D FLD in different views is given in Figure 5. Failure occurred in a critical element at a near plane strain state of $\varepsilon_{1}=0.26,\varepsilon_{2}=-0.045$ ($\eta =0.52,{\bar{\varepsilon }}_{p}^{f}=0.278$) and a temperature of $T_{blank}^{f}=186.9{\;}^{\circ }$C.

### 5.2. Johnson–Cook Based Approach

The second study investigates the forming limits of the given example by a combination of the generalized Johnson–Cook approach presented by Buyuk [30] from Equation (12) with the Barlat89-2D yield locus as described in Section 2. In analogy to the method proposed by Buyuk [30], the temperature dependent failure surface is created by multiplying the baseline failure envelope at room temperature by the stress state invariant temperature scaling function $h\left[T\right]={\bar{\varepsilon }}_{p}^{f}[\eta =1/3,T>25{\;}^{\circ }$C] ÷ ${\bar{\varepsilon }}_{p}^{f}[\eta =1/3,T=25{\;}^{\circ }$C].

**Figure 6:**
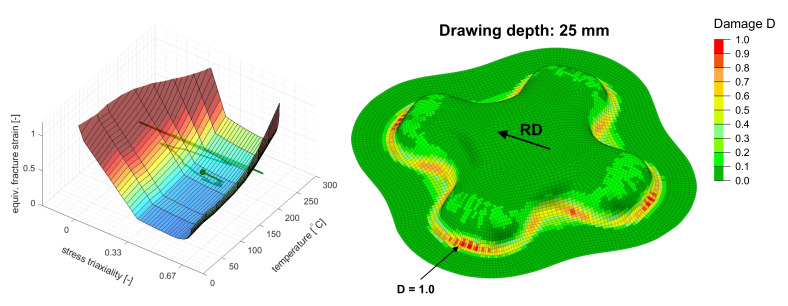
(**Left**): Underlying failure surface with the load path of the critical element; the predicted onset of necking is flagged as a dot. (**Right**): Contour plot of damage *D* (here necking indicator) at the inner surface of the deep-drawn cross-die cup at the predicted onset of necking.

**Figure 7:**
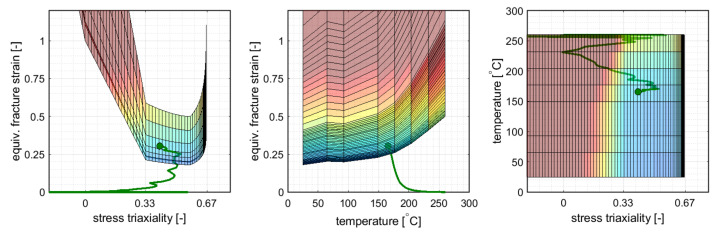
The failure surface of the JC-like approach at the onset of necking from Figure 6 depicted in different views.

The occurrence of necking was predicted in the transverse direction at the ends of the cross-die arms at a drawing depth of 25 mm. The underlying fracture surface and the load path of the critical element as well as the cross-die cup at the critical drawing depth are depicted in Figure 6. The failure surface and the load path of the critical element are depicted in Figure 7 in different representations. The predicted occurrence of necking was flagged as a dot. Failure occurred in a critical element at a stress state between uniaxial tension and plane strain of η=0.41 and an equivalent stain of ${\bar{\varepsilon }}_{p}^{f}=0.305$. The temperature at failure was $T_{blank}^{f}=165.7{\;}^{\circ }$C.

### 5.3. GISSMO with Temperature Effects

Within this numerical study the GISSMO fracture framework was used as an instability indicator. Thus, the scalar value *D* was to be interpreted as the onset of localized necking and not as the risk of material separation. Coupling between damage and stress as given by Equation (19) was deactivated for the purpose of this study. The damage exponent was set to n=1 which is in line with the original formulation of Johnson and Cook [29]. Therefore, the damage was accumulated linearly. In this way a direct comparison between a stress state independent (Section 5.2) and a stress state dependent temperature scaling (this section) was possible.

**Figure 8:**
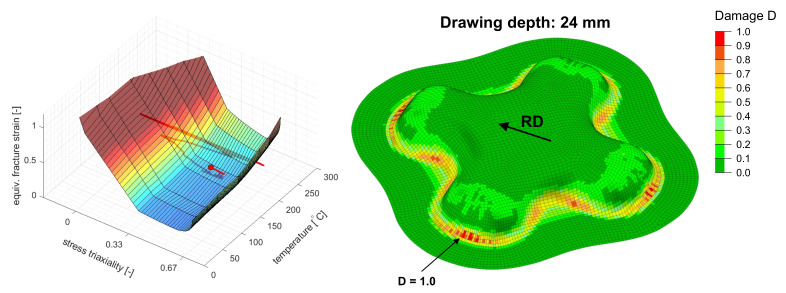
(**Left**): Underlying failure surface with the load path of the critical element; the predicted onset of necking is flagged as a red dot. (**Right**): Contour plot of damage *D* (here necking indicator) at the inner surface of the deep-drawn cross-die cup at the predicted onset of necking.

**Figure 9:**
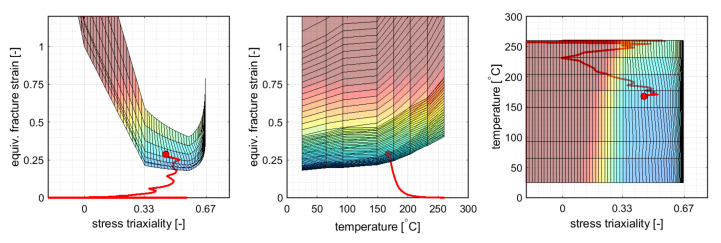
The failure surface at the onset of necking from Figure 8 depicted in different views.

The occurrence of necking was predicted in the transverse direction at the ends of the cross-die arms at a drawing depth of 24 mm. Figure 8 shows the cross-die cup at the critical drawing depth as well as the underlying fracture surface derived from a linear interpolation between data from Figure 2 with a plot of the critical element load path. The predicted occurrence of necking was flagged as a dot at the end of the load path. Figure 9 shows the failure surface and the load path of the critical element in different views. The GISSMO approach predicted necking at stress state between uniaxial tension and plane strain of η=0.45 and an equivalent stain of ${\bar{\varepsilon }}_{p}^{f}=0.288$. The temperature at failure was $T_{blank}^{f}=167.6{\;}^{\circ }$C.

## 6. Discussion

All three approaches predict the onset of necking at same geometry feature of the double symmetric cross-die which concurrently represents the blank zone with the highest temperature gradient. Nevertheless, the failure locus changes with respect to rolling direction between FLD and the differential approaches. The lowest drawability of 20 mm is predicted with the 3D FLD method at rolling direction followed by the temperature enhanced GISSMO failure indicator framework which predicts a drawability of 24 mm (+20%). The failure is predicted by GISSMO at transverse direction. The JC-like approach predicts a 25% (25 mm) higher draw depth in comparison with the 3D FLD method. Like GISSMO, the JC-like approach predicts the occurrence of failure in the transverse direction. The temperature at failure in the two later approaches is about 20 ${}^{\circ }$C (−12%) lower than the critical temperature in the 3D FLC method. The higher formability predictions of the JC-like and the GISSMO evaluation can be attributed to the incremental evaluation and accumulation of damage by taking into account the equivalent fracture strain at the current stress state and temperature, whereas in the 3D FLD approach the current element strain and temperature are evaluated against a static 3D FLC by neglecting the loading history. Consequently, material points that are pre-strained at higher temperatures and cooled down to lower temperatures with lower ductility could spuriously be interpreted as failed in the 3D FLD. This drawback vanishes with the incremental and temperature dependent damage accumulation approaches used in Section 5.2 and Section 5.3. From Figure 7 and Figure 9 it is apparent that necking occurs far beyond the limiting failure surface which is due to the correct employment of prior straining at higher temperatures.

**Figure 10:**
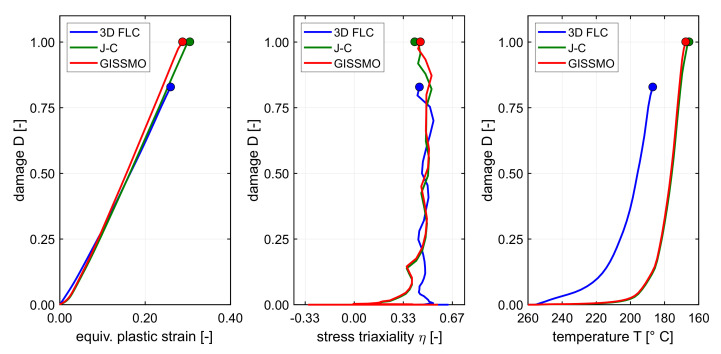
Loading paths of the critical elements from the 3D FLD, JC-like and GISSMO evaluation. For comparison purposes the loading path of the critical element from the 3D FLD is evaluated by damage *D* accumulation as given in Equation (10). The predicted onsets of necking are depicted by dots.

The loading paths of the critical elements with respect to distinctive state variables are depicted in Figure 10. For the purpose of comparison, the loading path of the critical element from the 3D FLD is evaluated by damage accumulation *D* as given in Equation (10). Likewise the drawing depth, the damage of the critical element from the 3D FLD is about 20% lower than this evaluated with the temperature enhanced GISSMO. Furthermore, Figure 10 shows that the strain path of the critical elements can be fairly assumed to be linear in all three cases, so that at least with respect to strain, an FLC evaluation assuming strain path proportionality should provide a sufficiently good prediction of the critical drawing depth. Contrarily, the temperature paths of the critical elements show a severe non-linearity which makes an incremental evaluation indispensable.

**Figure 11:**
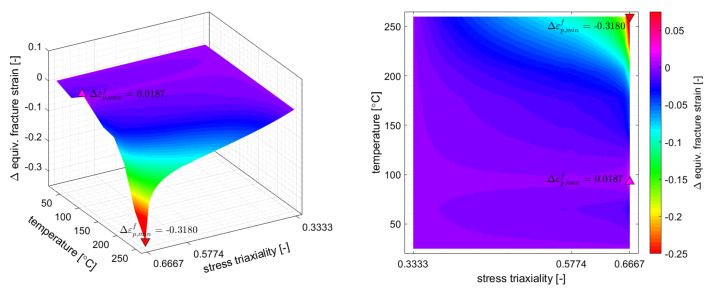
Difference between the predicted failure strain of the JC-like approach and the actual temperature dependent FLC data used by the GISSMO approach in the sheet metal forming relevant *“biaxial strain valley”* {η∣1/3≤η≤2/3}.

In order to quantify the difference between the stress state invariant temperature scaling of the baseline failure curve, on which the JC-like approach is based, and the actual forming limits of the investigated sheet material used in GISSMO, the difference between both failure strains is plotted in the sheet metal forming relevant *“biaxial tension valley”*, {η∣1/3≤η≤2/3}. The differential plot presented in Figure 11 shows that the overestimation of the JC-based assumption becomes larger with higher triaxialities and temperatures and amount up to $\Delta {\bar{\varepsilon }}_{p}^{f}=0.32$ with respect to the actual forming limit. For low triaxialities and temperatures the differences are negligible and approximately $\Delta {\bar{\varepsilon }}_{p}^{f}=\pm 0.02$. In turn, in applications dominated by uniaxial tension (η=1/3), the JC-like method will provide a decent prediction of the actual forming limit. In applications at higher temperatures dominated by biaxial tension (η=2/3), as for example non-isothermal sheet metal forming, a simple scaling of the baseline failure curve by a fixed ratio of ${\bar{\varepsilon }}_{p}^{f}[\eta =1/3,T>25{\;}^{\circ }$C] ÷ ${\bar{\varepsilon }}_{p}^{f}[\eta =1/3,T=25{\;}^{\circ }$C] will lead to significant deviations between the predicted and the actual failure strain given by FLCs.

These results illustrate the importance of taking into account the strain and temperature paths as well as the consideration of all available data regarding the forming limits of the investigated material when evaluating the formability in heat-assisted sheet metal forming processes.

## 7. Conclusions

An enrichment of the widely used GISSMO fracture indicator framework Andrade et al. [21] by temperature effects has been proposed to predict the onset of necking in heat-assisted forming processes. The enhancement is based on the pioneering work of Johnson and Cook [29] in which damage depends on the equivalent plastic strain to fracture under the current conditions of stress state, strain rate and temperature. By implementing a temperature dependency of the equivalent plastic fracture and equivalent plastic instability strain into the GISSMO fracture indicator framework, the instability as well as the damage indicator become temperature dependent and account for both non-linear strain and temperature paths.

The abilities of the temperature-dependent GISSMO model to predict the onset of necking are assessed by a numerical warm forming study and compared with the predictions of a JC-like model and a 3D FLD as presented by Buyuk [30] and Cui et al. [18], respectively. By taking the temperature path into account, GISSMO predicts a greater drawing depth by 20% than the 3D FLD. Moreover, the risk and locus of failure can be evaluated directly on the part geometry by a contour plot. An additional advantage of the GISSMO model is the applicability for low triaxialities as well as the possibility to predict the materials behavior beyond necking up to ductile fracture. The compatibility of GISSMO with arbitrary failure envelopes allows to adopt, e.g., temperature dependent FLC data, and in this way to evaluate the forming limits of sheet metal under non-isothermal conditions. This leads, compared with a JC-like approach, to more precise predictions of the actual forming limits given by experimental results or numerical models like the M-K model.

The phenomenology of ductile failure at elevated temperatures is a complex process influenced by strain rate changes as well as non-linear strain and temperature paths. An elaborate experimental program is in need to justify and validate the here proposed method. Furthermore, in the case of inconsistent mesh densities, a regularization scheme must be considered and investigated [21]. This is the subject of ongoing research.

## Figures and Tables

**Table 1:** Temperature dependent material parameters of Equation (1) adapted from Abedrabbo et al. [11].

*T*	K[T] in MPa	$n_{k}\left[T\right]$	$m_{k}\left[T\right]$
≤93 ${}^{\circ }$C	551.2–0.4623 ·T	0.3135–0.000363 ·T	0.00106·exp(0.01743·T)
>93 ${}^{\circ }$C	641.3–1.829 ·T	0.3687–0.001065 ·T	0.00106·exp(0.01743·T)

**Table 2:** Temperature dependent R-values adapted from Abedrabbo et al. [11].

	25 ${}^{\circ }$C	66 ${}^{\circ }$C	93 ${}^{\circ }$C	121 ${}^{\circ }$C	148 ${}^{\circ }$C	177 ${}^{\circ }$C	204 ${}^{\circ }$C	232 ${}^{\circ }$C	260 ${}^{\circ }$C
$R_{0}$	0.8085	0.9193	0.9719	1.1244	1.0550	1.1315	1.1521	1.0687	1.0687
$R_{45}$	1.0865	1.0533	1.1359	1.1260	1.1650	1.2576	1.1381	1.3006	1.3412
$R_{90}$	0.9986	1.0754	1.0634	1.2488	1.2001	1.2377	1.2623	1.2007	1.2340

**Table 3:** Pressure-dependent interfacial heat transfer coefficient $h_{IHTC}=f\left[p\right]$ between the tooling and blank.

Contact Pressure *p* [MPa]	$h_{\mathrm{IHTC}}=f\left[p\right]$ [W/(m${}^{-2}\cdot$ K)]
0.5	2.6
2.5	3.17
5.0	3.76
7.5	4.24
10.0	4.61
12.5	4.89
15.0	5.09
17.5	5.22
≥20.0	5.23

**Table 4:** Thermal properties of blank and tool used in the FE simulation.

Property	Blank (AA5182-O)	Tooling (Steel)
Heat capacity [J/(kg · K)]	900.0	450.0
Thermal conductivity [W/(m · K)]	130.0	30.0
Density [kg/m${}^{3}$]	2780.0	7830.0
Initial temperature [${}^{\circ }$C]	260.0	25.0

## Data Availability

Restrictions apply to the availability of these data. Data was obtained from International Journal of Plasticity, 23, Nader Abedrabbo, Farhang Pourboghrat, John Carsley, Forming of AA5182-O and AA5754-O at elevated temperatures using coupled thermo-mechanical finite element models, 841–875, Copyright (2007), and are available at https://doi.org/10.1016/j.ijplas.2006.10.005 (accessed on 29 August 2021) with permission from Elsevier.

## Abbreviations

The following abbreviations are used in this manuscript:

AA	Aluminum Alloy
FLC	Forming Limit Curve
FLD	Forming Limit Diagram
FE	Finite Element
FEA	Finite Element Analysis
GISSMO	Generalized Incremental Stress State Dependent Damage Model
M-K	Marciniak–Kuczyński Model
ISO	International Organization for Standardization
CDM	Continuum Damage Mechanics
JC	Johnson and Cook Model
RD	Rolling Direction
